# *catena*-Poly[[[diiodidomercury(II)]-μ-*N*,*N*′-di-3-pyridylpyridine-2,6-dicarboxamide] dimethyl­formamide solvate]

**DOI:** 10.1107/S1600536808036581

**Published:** 2008-11-13

**Authors:** Li-Hua Huang, Jie Wu, Fang-Fang Pan

**Affiliations:** a Department of Chemistry, Zhengzhou University, Zhengzhou 450052, People’s Republic of China

## Abstract

In the title complex, {[HgI_2_(C_17_H_13_N_5_O_2_)]·C_3_H_7_NO}_*n*_, the Hg atom is coordinated by two I atoms and two N atoms from two different ligands in a distorted tetra­hedral environment. Hg atoms are bridged by *N*,*N*′-di-3-pyridylpyridine-2,6-dicarboxamide ligands, forming a helical chain running along the *a* axis.

## Related literature

For binuclear complexes of *N*,*N*′-bis­(pyridin-3-yl)-2,6-pyridine­dicarboxamide, see: Baer *et al.* (2002 ▶); Huang & Wu (2008 ▶); Qin *et al.* (2003 ▶).
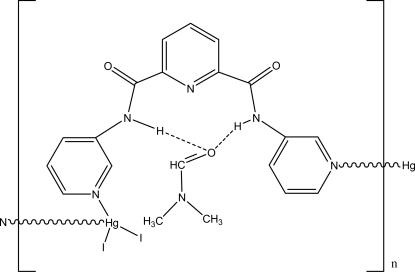


## Experimental

### 

#### Crystal data

[HgI_2_(C_17_H_13_N_5_O_2_)]·C_3_H_7_NO
*M* *_r_* = 846.81
Orthorhombic, 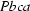

*a* = 21.295 (4) Å
*b* = 9.7177 (19) Å
*c* = 24.440 (5) Å
*V* = 5057.5 (17) Å^3^
*Z* = 8
Mo *K*α radiation
μ = 8.56 mm^−1^
*T* = 293 (2) K
0.20 × 0.18 × 0.08 mm

#### Data collection

Rigaku Saturn724 diffractometer
Absorption correction: numerical (*CrystalClear*; Rigaku/MSC, 2006 ▶) *T*_min_ = 0.279, *T*_max_ = 0.547
48014 measured reflections
4447 independent reflections
4250 reflections with *I* > 2σ(*I*)
*R*_int_ = 0.073

#### Refinement

*R*[*F* ^2^ > 2σ(*F* ^2^)] = 0.062
*wR*(*F* ^2^) = 0.119
*S* = 1.31
4447 reflections
291 parameters
H-atom parameters constrained
Δρ_max_ = 1.21 e Å^−3^
Δρ_min_ = −0.92 e Å^−3^

### 

Data collection: *CrystalClear* (Rigaku/MSC, 2006 ▶); cell refinement: *CrystalClear*; data reduction: *CrystalClear*; program(s) used to solve structure: *SHELXS97* (Sheldrick, 2008 ▶); program(s) used to refine structure: *SHELXL97* (Sheldrick, 2008 ▶); molecular graphics: *SHELXTL* (Sheldrick, 2008 ▶); software used to prepare material for publication: *SHELXTL*.

## Supplementary Material

Crystal structure: contains datablocks global, I. DOI: 10.1107/S1600536808036581/hg2428sup1.cif

Structure factors: contains datablocks I. DOI: 10.1107/S1600536808036581/hg2428Isup2.hkl

Additional supplementary materials:  crystallographic information; 3D view; checkCIF report

## Figures and Tables

**Table 1:** Hydrogen-bond geometry (Å, °)

*D*—H⋯*A*	*D*—H	H⋯*A*	*D*⋯*A*	*D*—H⋯*A*
N4—H4⋯O3	0.86	2.23	3.008 (10)	150
N2—H2⋯O3	0.86	2.26	2.964 (10)	139

**Fig. 1.**
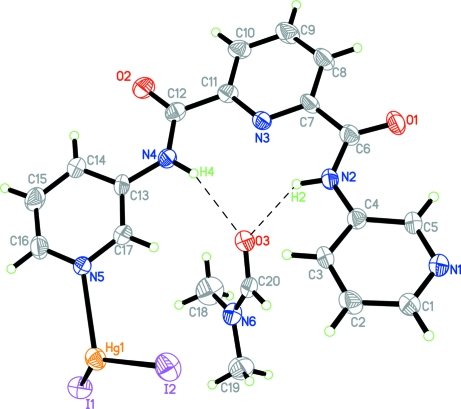
View of the title complex, showing the labeling of the non-H atoms and 30% probability ellipsolids.

**Fig. 2.**
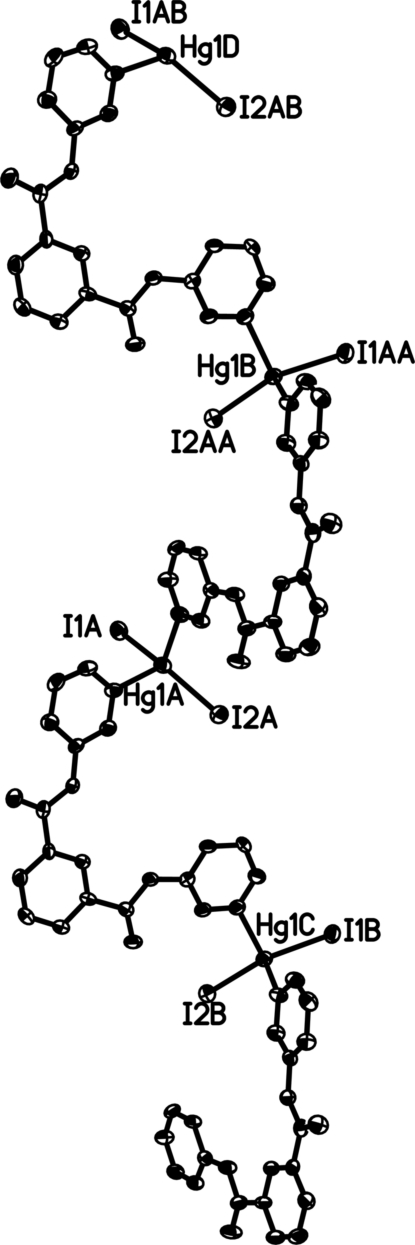
A view of the crystal packing along the c axis.

## supplementary crystallographic information

### Comment

The expansion of the field of metal-organic frameworks (MOFs) of predetermined structure depends on the judicious choice of new linkers and nodes of appropriate coordination algorithms. Rigid, polydentate N-donor ligands are typical linkers employed in such work. The rigid conjugated clamp-like multi-pyridine ligand *N*,*N*'-bis(pyridin-3-yl)-2,6-pyridinedicarboxamide, is a convenient bridging ligand for the synthesis of chain complexes. However, the previous work proved that the ligand also can form binuclear complex with 28-number ring (Huang *et al.*; 2004, Qin *et al.* 2003; Baer *et al.* 2002). We think, among the factors that induce the self-assembly processes with this ligand, the rotation of terminal pyridine groups plays a crucial role in deciding between binuclear *versus*. extended structures of the metal complexes. In this work, we selected this ligand as linker, generating a new helical chain coordination complex, {[HgI_2_(C_17_N_5_O_2_)](DMF)}_n_, (I), which is reported here. In compound (I) each Hg(II) atom is four-coordinated by two N atoms from two ligands and two I atoms in a distorted tetrahedral coordination sphere (Fig. 1). The Hg(II) atoms are bridged with *N*,*N*'-bis(pyridin-3-yl)-2,6-pyridinedicarboxamide ligands to form a helical chain running the *a* axis (Fig. 2).

In the crystal structure, the intermolecular N—H···N hydrogen bonds and the N—H···O hydrogen bonds arising from the DMF and ligand complete the structure.

### Experimental

The ligand *N*,*N*'-bis(pyridin-3-yl)-2,6-pyridinedicarboxamide (0.05 mmol, 0.016 g) in DMF (5 ml) was added dropwise to a solution of HgI2 (0.1 mmol, 0.036 g) in methanol (3 ml). The precipitate was filtered and the resulting solution was allowed to stand at room temperature in the dark. After one week good quality colorless crystals were obtained from the filtrate and dried in air.

### Crystal data

**Table:**

[HgI_2_(C_17_H_13_N_5_O_2_)]·C_3_H_7_NO	*F*_000_ = 3136
*M**_r_* = 846.81	*D*_x_ = 2.224 Mg m^−^^3^
Orthorhombic, *P**b**c**a*	Mo *K*α radiation λ = 0.71073 Å
Hall symbol: -P 2ac 2ab	Cell parameters from 8721 reflections
*a* = 21.295 (4) Å	θ = 3.2–26.0º
*b* = 9.7177 (19) Å	µ = 8.56 mm^−^^1^
*c* = 24.440 (5) Å	*T* = 293 (2) K
*V* = 5057.5 (17) Å^3^	Prism, colorless
*Z* = 8	0.20 × 0.18 × 0.08 mm

### Data collection

**Table:**

Saturn724 diffractometer	4447 independent reflections
Radiation source: fine-focus sealed tube	4250 reflections with *I* > 2σ(*I*)
Monochromator: graphite	*R*_int_ = 0.073
Detector resolution: 28.5714 pixels mm^-1^	θ_max_ = 25.0º
*T* = 291(2) K	θ_min_ = 3.3º
dtprofit.ref scans	*h* = −25→25
Absorption correction: Numerical(CrystalClear; Rigaku/MSC, 2006)	*k* = −11→11
*T*_min_ = 0.279, *T*_max_ = 0.548	*l* = −29→29
48014 measured reflections	

### Refinement

**Table:**

Refinement on *F*^2^	Secondary atom site location: difference Fourier map
Least-squares matrix: full	Hydrogen site location: inferred from neighbouring sites
*R*[*F*^2^ > 2σ(*F*^2^)] = 0.062	H-atom parameters constrained
*wR*(*F*^2^) = 0.119	*w* = 1/[σ^2^(*F*_o_^2^) + (0.0319*P*)^2^ + 27.0127*P*] where *P* = (*F*_o_^2^ + 2*F*_c_^2^)/3
*S* = 1.31	(Δ/σ)_max_ = 0.001
4447 reflections	Δρ_max_ = 1.21 e Å^−^^3^
291 parameters	Δρ_min_ = −0.92 e Å^−^^3^
Primary atom site location: structure-invariant direct methods	Extinction correction: none

### Special details

**Table:**

Geometry. All e.s.d.'s (except the e.s.d. in the dihedral angle between two l.s. planes) are estimated using the full covariance matrix. The cell e.s.d.'s are taken into account individually in the estimation of e.s.d.'s in distances, angles and torsion angles; correlations between e.s.d.'s in cell parameters are only used when they are defined by crystal symmetry. An approximate (isotropic) treatment of cell e.s.d.'s is used for estimating e.s.d.'s involving l.s. planes.
Refinement. Refinement of *F*^2^ against ALL reflections. The weighted *R*-factor *wR* and goodness of fit *S* are based on *F*^2^, conventional *R*-factors *R* are based on *F*, with *F* set to zero for negative *F*^2^. The threshold expression of *F*^2^ > σ(*F*^2^) is used only for calculating *R*-factors(gt) *etc*. and is not relevant to the choice of reflections for refinement. *R*-factors based on *F*^2^ are statistically about twice as large as those based on *F*, and *R*- factors based on ALL data will be even larger.

### Fractional atomic coordinates and isotropic or equivalent isotropic displacement parameters (Å^2^)

**Table:**

	*x*	*y*	*z*	*U*_iso_*/*U*_eq_	
Hg1	0.374277 (19)	0.12855 (4)	0.670365 (18)	0.05404 (16)	
I1	0.34375 (4)	−0.02626 (8)	0.58368 (3)	0.0644 (2)	
I2	0.44953 (4)	0.13315 (9)	0.75677 (3)	0.0709 (3)	
O1	0.8018 (3)	0.4961 (8)	0.6739 (3)	0.069 (2)	
O2	0.5375 (3)	0.7089 (8)	0.5249 (3)	0.065 (2)	
O3	0.5923 (3)	0.2832 (8)	0.6383 (3)	0.063 (2)	
N1	0.7739 (4)	0.2015 (9)	0.7954 (4)	0.053 (2)	
N2	0.7026 (3)	0.4172 (9)	0.6896 (3)	0.050 (2)	
H2	0.6649	0.4223	0.6772	0.060*	
N3	0.6581 (3)	0.5688 (8)	0.6070 (3)	0.0432 (18)	
N4	0.5345 (3)	0.5356 (8)	0.5879 (3)	0.0450 (19)	
H4	0.5581	0.4874	0.6091	0.054*	
N5	0.3933 (4)	0.3479 (7)	0.6244 (4)	0.047 (2)	
N6	0.5553 (4)	0.0721 (9)	0.6163 (4)	0.053 (2)	
C1	0.7230 (5)	0.1535 (11)	0.8191 (5)	0.059 (3)	
H1	0.7274	0.0923	0.8481	0.070*	
C2	0.6634 (5)	0.1903 (12)	0.8027 (4)	0.056 (3)	
H2A	0.6281	0.1551	0.8202	0.067*	
C3	0.6576 (4)	0.2814 (10)	0.7594 (4)	0.048 (2)	
H5	0.6181	0.3091	0.7473	0.057*	
C4	0.7112 (4)	0.3301 (10)	0.7344 (4)	0.044 (2)	
C5	0.7690 (4)	0.2899 (10)	0.7538 (4)	0.049 (2)	
H3	0.8053	0.3247	0.7377	0.059*	
C6	0.7461 (5)	0.4951 (11)	0.6630 (4)	0.051 (3)	
C7	0.7196 (4)	0.5800 (10)	0.6170 (4)	0.048 (2)	
C8	0.7579 (5)	0.6690 (12)	0.5882 (5)	0.061 (3)	
H8	0.8005	0.6756	0.5962	0.073*	
C9	0.7315 (5)	0.7476 (13)	0.5473 (5)	0.067 (3)	
H9	0.7564	0.8069	0.5268	0.081*	
C10	0.6680 (5)	0.7383 (11)	0.5367 (4)	0.057 (3)	
H10	0.6493	0.7922	0.5099	0.068*	
C11	0.6331 (4)	0.6463 (10)	0.5674 (4)	0.044 (2)	
C12	0.5642 (5)	0.6341 (10)	0.5573 (4)	0.048 (2)	
C13	0.4705 (4)	0.5039 (9)	0.5891 (4)	0.042 (2)	
C14	0.4245 (5)	0.5696 (11)	0.5587 (5)	0.054 (3)	
H14	0.4345	0.6445	0.5367	0.065*	
C15	0.3633 (5)	0.5216 (12)	0.5618 (5)	0.063 (3)	
H15	0.3314	0.5648	0.5422	0.075*	
C16	0.3505 (5)	0.4110 (12)	0.5937 (5)	0.059 (3)	
H16	0.3096	0.3774	0.5942	0.071*	
C17	0.4519 (5)	0.3943 (10)	0.6222 (4)	0.050 (3)	
H17	0.4820	0.3516	0.6439	0.061*	
C18	0.5424 (7)	0.1046 (15)	0.5597 (5)	0.091 (4)	
H18A	0.4990	0.0863	0.5519	0.136*	
H18B	0.5683	0.0489	0.5365	0.136*	
H18C	0.5511	0.2001	0.5532	0.136*	
C19	0.5445 (6)	−0.0682 (12)	0.6371 (6)	0.081 (4)	
H19A	0.5770	−0.1281	0.6239	0.121*	
H19B	0.5045	−0.1008	0.6245	0.121*	
H19C	0.5450	−0.0672	0.6763	0.121*	
C20	0.5801 (4)	0.1635 (10)	0.6498 (4)	0.047 (2)	
H20	0.5891	0.1345	0.6852	0.057*	

### Atomic displacement parameters (Å^2^)

**Table:**

	*U*^11^	*U*^22^	*U*^33^	*U*^12^	*U*^13^	*U*^23^
Hg1	0.0468 (3)	0.0576 (3)	0.0577 (3)	−0.00017 (19)	0.00731 (19)	−0.0057 (2)
I1	0.0753 (5)	0.0618 (5)	0.0562 (5)	−0.0038 (4)	0.0088 (4)	−0.0071 (4)
I2	0.0654 (5)	0.0874 (6)	0.0599 (5)	−0.0060 (4)	−0.0026 (4)	−0.0047 (4)
O1	0.039 (4)	0.085 (6)	0.083 (6)	−0.017 (4)	−0.016 (4)	0.029 (4)
O2	0.068 (5)	0.063 (5)	0.066 (5)	−0.002 (4)	−0.015 (4)	0.024 (4)
O3	0.047 (4)	0.058 (5)	0.084 (6)	−0.006 (4)	−0.013 (4)	0.012 (4)
N1	0.045 (5)	0.055 (5)	0.058 (6)	−0.002 (4)	−0.011 (4)	0.010 (4)
N2	0.033 (4)	0.057 (5)	0.060 (5)	−0.003 (4)	−0.007 (4)	0.017 (4)
N3	0.040 (4)	0.044 (4)	0.046 (5)	−0.005 (4)	0.000 (4)	0.003 (4)
N4	0.044 (4)	0.051 (5)	0.040 (5)	0.000 (4)	−0.007 (4)	0.013 (4)
N5	0.038 (4)	0.037 (4)	0.066 (6)	0.000 (3)	−0.007 (4)	0.003 (4)
N6	0.049 (5)	0.055 (5)	0.054 (6)	0.008 (4)	−0.005 (4)	−0.003 (4)
C1	0.044 (6)	0.062 (7)	0.070 (8)	−0.005 (5)	−0.001 (5)	0.017 (6)
C2	0.045 (6)	0.076 (7)	0.046 (6)	−0.010 (5)	0.013 (5)	0.010 (6)
C3	0.029 (5)	0.055 (6)	0.060 (7)	−0.003 (4)	−0.003 (4)	0.004 (5)
C4	0.042 (5)	0.044 (5)	0.045 (6)	0.001 (4)	−0.005 (4)	−0.002 (4)
C5	0.036 (5)	0.054 (6)	0.058 (6)	0.000 (5)	−0.007 (5)	0.006 (5)
C6	0.042 (6)	0.059 (6)	0.051 (6)	−0.007 (5)	0.001 (5)	0.011 (5)
C7	0.035 (5)	0.055 (6)	0.055 (6)	−0.013 (5)	0.003 (4)	0.003 (5)
C8	0.044 (6)	0.076 (7)	0.062 (7)	−0.020 (5)	−0.002 (5)	0.014 (6)
C9	0.062 (7)	0.079 (8)	0.061 (7)	−0.022 (6)	0.006 (6)	0.022 (6)
C10	0.069 (7)	0.053 (6)	0.048 (6)	−0.013 (5)	−0.009 (5)	0.013 (5)
C11	0.053 (6)	0.049 (6)	0.030 (5)	−0.005 (5)	0.000 (4)	0.008 (4)
C12	0.059 (6)	0.048 (6)	0.037 (6)	0.003 (5)	−0.005 (5)	0.005 (5)
C13	0.035 (5)	0.047 (5)	0.043 (5)	0.008 (4)	−0.008 (4)	−0.004 (4)
C14	0.047 (6)	0.049 (6)	0.067 (7)	0.007 (5)	−0.003 (5)	0.006 (5)
C15	0.060 (7)	0.065 (7)	0.063 (8)	0.009 (6)	−0.020 (6)	0.008 (6)
C16	0.039 (5)	0.065 (7)	0.073 (8)	0.004 (5)	−0.007 (5)	−0.003 (6)
C17	0.045 (6)	0.047 (6)	0.060 (7)	0.005 (5)	−0.007 (5)	0.009 (5)
C18	0.110 (11)	0.111 (12)	0.052 (8)	0.000 (9)	−0.008 (7)	−0.003 (8)
C19	0.081 (9)	0.050 (7)	0.111 (11)	0.002 (6)	0.006 (8)	−0.011 (7)
C20	0.039 (5)	0.046 (6)	0.057 (6)	0.009 (5)	−0.005 (5)	0.003 (5)

### Geometric parameters (Å, °)

**Table:**

Hg1—N1^i^	2.403 (8)	C3—H5	0.9300
Hg1—N5	2.444 (8)	C4—C5	1.377 (12)
Hg1—I2	2.6514 (10)	C5—H3	0.9300
Hg1—I1	2.6785 (10)	C6—C7	1.504 (14)
O1—C6	1.215 (12)	C7—C8	1.381 (14)
O2—C12	1.217 (11)	C8—C9	1.380 (15)
O3—C20	1.225 (11)	C8—H8	0.9300
N1—C1	1.314 (13)	C9—C10	1.378 (14)
N1—C5	1.335 (12)	C9—H9	0.9300
N1—Hg1^ii^	2.403 (8)	C10—C11	1.384 (13)
N2—C6	1.362 (12)	C10—H10	0.9300
N2—C4	1.395 (12)	C11—C12	1.492 (13)
N2—H2	0.8600	C13—C14	1.387 (13)
N3—C7	1.336 (11)	C13—C17	1.395 (13)
N3—C11	1.337 (11)	C14—C15	1.387 (14)
N4—C12	1.370 (12)	C14—H14	0.9300
N4—C13	1.396 (11)	C15—C16	1.356 (16)
N4—H4	0.8600	C15—H15	0.9300
N5—C17	1.327 (12)	C16—H16	0.9300
N5—C16	1.330 (13)	C17—H17	0.9300
N6—C20	1.318 (13)	C18—H18A	0.9600
N6—C18	1.445 (15)	C18—H18B	0.9600
N6—C19	1.473 (14)	C18—H18C	0.9600
C1—C2	1.379 (14)	C19—H19A	0.9600
C1—H1	0.9300	C19—H19B	0.9600
C2—C3	1.386 (14)	C19—H19C	0.9600
C2—H2A	0.9300	C20—H20	0.9300
C3—C4	1.378 (13)		
			
N1^i^—Hg1—N5	92.9 (3)	C9—C8—H8	120.8
N1^i^—Hg1—I2	104.8 (2)	C7—C8—H8	120.8
N5—Hg1—I2	104.57 (19)	C10—C9—C8	119.9 (10)
N1^i^—Hg1—I1	103.0 (2)	C10—C9—H9	120.0
N5—Hg1—I1	99.6 (2)	C8—C9—H9	120.0
I2—Hg1—I1	141.83 (3)	C9—C10—C11	117.9 (10)
C1—N1—C5	120.1 (9)	C9—C10—H10	121.0
C1—N1—Hg1^ii^	118.3 (7)	C11—C10—H10	121.0
C5—N1—Hg1^ii^	121.6 (6)	N3—C11—C10	122.8 (9)
C6—N2—C4	128.5 (8)	N3—C11—C12	117.9 (8)
C6—N2—H2	115.7	C10—C11—C12	119.3 (9)
C4—N2—H2	115.7	O2—C12—N4	123.9 (9)
C7—N3—C11	118.5 (8)	O2—C12—C11	121.3 (9)
C12—N4—C13	128.1 (8)	N4—C12—C11	114.8 (8)
C12—N4—H4	116.0	C14—C13—C17	117.5 (9)
C13—N4—H4	116.0	C14—C13—N4	125.2 (9)
C17—N5—C16	117.8 (9)	C17—C13—N4	117.3 (8)
C17—N5—Hg1	118.0 (6)	C13—C14—C15	118.7 (10)
C16—N5—Hg1	123.2 (7)	C13—C14—H14	120.6
C20—N6—C18	121.6 (10)	C15—C14—H14	120.6
C20—N6—C19	118.2 (10)	C16—C15—C14	119.2 (10)
C18—N6—C19	120.1 (10)	C16—C15—H15	120.4
N1—C1—C2	122.6 (10)	C14—C15—H15	120.4
N1—C1—H1	118.7	N5—C16—C15	123.5 (10)
C2—C1—H1	118.7	N5—C16—H16	118.3
C1—C2—C3	118.1 (9)	C15—C16—H16	118.3
C1—C2—H2A	121.0	N5—C17—C13	123.3 (9)
C3—C2—H2A	121.0	N5—C17—H17	118.3
C4—C3—C2	119.0 (9)	C13—C17—H17	118.3
C4—C3—H5	120.5	N6—C18—H18A	109.5
C2—C3—H5	120.5	N6—C18—H18B	109.5
C5—C4—C3	119.4 (9)	H18A—C18—H18B	109.5
C5—C4—N2	124.0 (9)	N6—C18—H18C	109.5
C3—C4—N2	116.6 (8)	H18A—C18—H18C	109.5
N1—C5—C4	120.9 (9)	H18B—C18—H18C	109.5
N1—C5—H3	119.5	N6—C19—H19A	109.5
C4—C5—H3	119.5	N6—C19—H19B	109.5
O1—C6—N2	124.3 (9)	H19A—C19—H19B	109.5
O1—C6—C7	121.7 (9)	N6—C19—H19C	109.5
N2—C6—C7	114.0 (8)	H19A—C19—H19C	109.5
N3—C7—C8	122.4 (10)	H19B—C19—H19C	109.5
N3—C7—C6	117.4 (8)	O3—C20—N6	125.7 (10)
C8—C7—C6	120.2 (9)	O3—C20—H20	117.2
C9—C8—C7	118.4 (10)	N6—C20—H20	117.2
			
N1^i^—Hg1—N5—C17	145.4 (8)	C6—C7—C8—C9	−178.4 (11)
I2—Hg1—N5—C17	39.3 (8)	C7—C8—C9—C10	1.3 (19)
I1—Hg1—N5—C17	−110.8 (7)	C8—C9—C10—C11	−1.5 (18)
N1^i^—Hg1—N5—C16	−46.3 (9)	C7—N3—C11—C10	−0.6 (15)
I2—Hg1—N5—C16	−152.4 (8)	C7—N3—C11—C12	−179.1 (9)
I1—Hg1—N5—C16	57.4 (8)	C9—C10—C11—N3	1.2 (16)
C5—N1—C1—C2	0.2 (17)	C9—C10—C11—C12	179.7 (10)
Hg1^ii^—N1—C1—C2	179.4 (9)	C13—N4—C12—O2	−1.3 (17)
N1—C1—C2—C3	0.2 (17)	C13—N4—C12—C11	177.4 (9)
C1—C2—C3—C4	0.5 (16)	N3—C11—C12—O2	174.3 (10)
C2—C3—C4—C5	−1.5 (15)	C10—C11—C12—O2	−4.3 (15)
C2—C3—C4—N2	177.5 (9)	N3—C11—C12—N4	−4.4 (13)
C6—N2—C4—C5	−11.9 (17)	C10—C11—C12—N4	177.0 (9)
C6—N2—C4—C3	169.2 (10)	C12—N4—C13—C14	−0.5 (16)
C1—N1—C5—C4	−1.2 (15)	C12—N4—C13—C17	177.3 (10)
Hg1^ii^—N1—C5—C4	179.6 (7)	C17—C13—C14—C15	−1.4 (15)
C3—C4—C5—N1	1.9 (15)	N4—C13—C14—C15	176.4 (10)
N2—C4—C5—N1	−177.1 (9)	C13—C14—C15—C16	−1.0 (17)
C4—N2—C6—O1	2.9 (18)	C17—N5—C16—C15	−1.8 (17)
C4—N2—C6—C7	−178.4 (9)	Hg1—N5—C16—C15	−170.1 (9)
C11—N3—C7—C8	0.3 (15)	C14—C15—C16—N5	2.7 (19)
C11—N3—C7—C6	178.0 (9)	C16—N5—C17—C13	−0.9 (15)
O1—C6—C7—N3	177.9 (10)	Hg1—N5—C17—C13	168.1 (8)
N2—C6—C7—N3	−0.9 (14)	C14—C13—C17—N5	2.4 (15)
O1—C6—C7—C8	−4.3 (17)	N4—C13—C17—N5	−175.6 (9)
N2—C6—C7—C8	176.9 (10)	C18—N6—C20—O3	3.1 (16)
N3—C7—C8—C9	−0.7 (18)	C19—N6—C20—O3	179.3 (10)

Symmetry codes: (i) *x*−1/2, *y*, −*z*+3/2; (ii) *x*+1/2, *y*, −*z*+3/2.

### Hydrogen-bond geometry (Å, °)

**Table:**

*D*—H···*A*	*D*—H	H···*A*	*D*···*A*	*D*—H···*A*
N4—H4···O3	0.86	2.23	3.008 (10)	150
N2—H2···O3	0.86	2.26	2.964 (10)	139
